# Effects of childhood trauma on nonsuicidal self‐injury in adolescent patients with bipolar II depression

**DOI:** 10.1002/brb3.2771

**Published:** 2022-09-28

**Authors:** Yanyan Zhang, Zhizhong Hu, Maorong Hu, Zihang Lu, Huijuan Yu, Xin Yuan

**Affiliations:** ^1^ Department of Psychosomatic Medicine The First Affiliated Hospital of Nanchang University Nanchang Jiangxi Province China; ^2^ School of Public Administration Nanchang University Nanchang Jiangxi Province China

**Keywords:** alexithymia, bipolar disorder, childhood trauma, nonsuicidal self‐injury

## Abstract

**Objective:**

This study was performed to explore the effect of childhood trauma on nonsuicidal self‐injury (NSSI) in adolescents with bipolar II (BD II) depression.

**Methods:**

Based on the diagnostic criteria of the DSM‐5 and structured interviews to assess the presence or absence of NSSI, 184 adolescent patients with BD II depression were divided into the NSSI (*n* = 112) and non‐NSSI (*n* = 72) groups. The Adolescent Nonsuicidal Self‐Injury Assessment Questionnaire (ANSAQ), Childhood Trauma Questionnaire‑Short Form (CTQ‐SF), Toronto Alexithymia Scale (TAS‐20), Hamilton Depression Scale (HAMD), and Hamilton Anxiety Scale (HAMA) were used to assess the subjects.

**Result:**

The CTQ‐SF, HAMD, HAMA, and TAS‐20 scores were significantly higher in the NSSI group than in the non‐NSSI group (*p* < .01). Logistic regression analysis showed emotional abuse (*p* = .028, OR = 1.14, 95% CI = 1.01–1.28) and age of onset (*p* = .009) as risk factors for NSSI. Adolescents with onset age 12–13 years (OR = 6.30, 95% CI = 1.72–23.10) and 14–15 years (OR = 2.24, 95% CI = 1.04–4.84) had a higher risk of self‐injury relative to adolescents aged 16–18 years.

**Conclusion:**

Childhood trauma and alexithymia were important influencing factors in adolescent patients with BD II depression. Emotional abuse and age of onset as risk factors for NSSI, and difficulties in emotion recognition were positively associated with the patients’ NSSI.

## Graphical abstract

We investigated the role of the a prominent serotonin transporter gene polymorphism for autistic traits and internet use disorder. Molecular genetic associations with autistic traits could be observed, but no associations with Internet Use Disorders appeared.

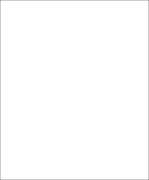

## 1 INTRODUCTION

Nonsuicidal self‐injury (NSSI) is an act of intentionally injuring oneself for 5 or more days within the past 12 months. This act is not suicidal in intent and socially and culturally unaccepted (International Society for the Study of Self Injury, 2018). NSSI is a prominent psychiatric problem among adolescents worldwide and a red flag that predicts suicidal thoughts and behaviors (Ribeiro et al., 2016). The study found that adolescents at risk for suicidal behavior may search more often on the Internet for information about self‐injury and suicide (Solano et al., 2016). A recent study has shown that the lifetime prevalence of NSSI among children and adolescents is 22.1% (Lim et al., 2019). One of the largest studies of NSSI in adolescents in Sweden has reported that 35.6% of adolescents had experienced this behavior at least once (Zetterqvist et al., 2013). NSSI is present in approximately 11.5%–41.5% of adolescents in China (Kang et al., 2018; Wang et al., 2016). Previous studies have demonstrated a strong association between NSSI and bipolar disorder (BD), with approximately 52% of patients with BD having been involved in at least one NSSI (Weintraub et al., 2017)

BD is characterized by recurrent episodes of hypomania, mania, or depression (Diagnostic and Statistical Manual of Mental Disorders, Fifth edition [DSM‐5], 2013). BD is divided into BD I, which is associated with mania, and BD II, which is associated with hypomania. BD usually causes concern because of depressive episodes and the duration of the depressive state is longer than manic and mixed states; therefore, bipolar depression is often misdiagnosed as unipolar depression (Chen et al., 2019; Miller et al., 2014). However, studies have shown that adolescents with bipolar depression have more documented abnormalities in the brain than those with unipolar depression (Serafini et al., 2014).Although previous studies have found that single nucleotide polymorphisms (SNPs) are associated with the development of BD, the neurobiological mechanisms underlying the BD pathology are unclear (Kerner, 2014). The pathogenesis of BD from the perspective of gene–environment interaction models consists of three main components, namely, genome, epigenetic regulation, and environmental factors (Rybakowski, 2017).

Childhood trauma has been reported to possibly reduce the age of onset in patients with BD and is an important environmental factor influencing the pathogenesis and course of such disorder (Sun et al., 2022). A possible strong association between childhood trauma and NSSI has been reported (Thomassin et al., 2016). Childhood trauma may be closely related to the hypothalamic–pituitary–adrenal axis, serotonergic signaling, and the amygdala (Aas et al., 2016; Paquola et al., 2016). Neuroimaging studies have suggested possible alterations in the frontal–limbic circuits, especially in the amygdala, which is responsible for negative emotion processing. Patients with NSSI tend to have strong emotional reactions and lack emotion regulation skills (Lindquist et al., 2016; Tatnell et al., 2017). The above studies help to better understand the mechanism of the link between childhood trauma and NSSI.

Related studies support the significant influence of childhood trauma and alexithymia on adolescent NSSI. However, different studies have obtained different results on the specific factor that influences variables of childhood trauma and alexithymia (Moseley et al., 2019; Norman & Borrill, 2015; Thomassin et al., 2016). Furthermore, research on factors influencing NSSI in adolescent patients with BD II depression is lacking. Thus, this study investigated the relationship between childhood trauma and NSSI in adolescents with BD II depression, using a sample of adolescents with BD II depression. The following hypotheses are proposed:

1. Demographic differences between the NSSI and nonNSSI groups regarding sex, only child, residence, and age of onset.
2. Childhood trauma, alexithymia, depression, anxiety and NSSI were significantly associated.
3. Emotional abuse as a risk factor for NSSI.

## 2 MATERIALS AND METHODS

### 2.1 Participants

Patients with first‐onset adolescent BD II depression who were seen in the outpatient and inpatient departments of the First Affiliated Hospital of Nanchang University from March 2020 to March 2022 were selected. The patients with BD II depression were diagnosed by experienced psychiatrists based on the DSM‐5. Inclusion criteria were as follows: (1) aged 12–18 years; (2) first visit without any history of medication or psychotherapy; (3) comfortable communication with the study staff; and (4) informed consent. Exclusion criteria were as follows: (1) known structural brain/neurological comorbidities; (2) schizophrenia and obsessive‐compulsive disorder; and (3) alcohol, drug, and substance dependence.

The study was approved by the Ethics Committee of the First Affiliated Hospital of Nanchang University. The subjects were truthfully informed of the content and purpose of the study, and their informed consent was obtained.

### 2.2 Methods

#### 2.2.1 Diagnosis of NSSI

Based on the DSM‐5 diagnostic criteria and structured interviews by two trained psychiatrists, the subjects diagnosed with NSSI were included in the NSSI group, and the rest in the non‐NSSI group.

#### 2.2.2 Research tools

##### Adolescent Nonsuicidal Self‐Injury Assessment Questionnaire (ANSAQ) developed by Wan et al. (2018)

This tool contains two dimensions, namely, behavioral and functional questionnaires, with 12 items on the behavioral questionnaire and 19 items on the functional questionnaire. A five‐point Likert scale (from 1[never] to 5[always]) was used, with higher scores indicating more severe self‐injury. The Cronbach's α coefficients for the behavioral and functional questionnaires in this study were .92 and .91, respectively.

##### Childhood Trauma Questionnaire‑Short Form (CTQ‑SF) (The Chinese version of the CTQ‐SF; Zhao et al., 2005)

This form includes five subscales of physical abuse, sexual abuse, emotional abuse, somatic neglect, and emotional neglect, with 28 entries, three of which are validity assessment entries. The questionnaire is scored on a five‐point Likert scale (from 1 [never] to 5 [always]), with higher scores indicating more severe trauma. The Cronbach's α coefficient for this scale in this study was .88.

##### Toronto Alexithymia Scale (TAS‐20)

The Chinese version of the TAS‐20 (Yi et al., 2003) contains three factors of difficulty in emotion recognition, difficulty in emotion description, and externally oriented thinking. This tool has a total of 20 entries. A five‐point Likert scale (from 1 [completely disagree] to 5 [completely agree]) was used, with higher scores indicating a greater degree of affective description difficulties. The Cronbach's α coefficient for this scale in this study was .69.

##### Hamilton Depression Scale (HAMD)

This scale consists of five components, namely, anxiety/somatization, weight, cognitive impairment, impairment, and sleep disturbance. HAMD has a total of 24 entries and scored using a 5‐point Likert scale, with higher total scores indicating more severe depression.

##### Hamilton Anxiety Scale (HAMA)

This scale consists of two parts, namely, somatic anxiety and mental anxiety. HAMA has a total of 14 items and scored using a 5‐point Likert scale. The higher the total score, the more severe the anxiety was.

### 2.3 Statistical analysis

SPSS 26.0 software was used for the statistical analysis. The χ^2^ and *t*‐test were used to compare the two groups on demographic characteristics and CTQ‐SF, HAMD, HAMA, and TAS‐20, respectively. Correlation analysis was used to explore the correlation between ANSAQ, CTQ‐SF, TAS‐20, HAMD, and HAMA. Logistic regression analysis was performed to explore the risk factors for NSSI. The test level was *p* = .05.

## 3 RESULTS

### 3.1 Demographic characteristics and comparison of CTQ‐SF, HAMD, HAMA, and TAS‐20 scores

The demographic characteristics and CTQ‐SF, HAMD, HAMA, and TAS‐20 scores in the concomitant NSSI and nonconcomitant NSSI groups are shown in Table 1. The age of onset variable shows a significant difference between the two groups. The age of onset in the concomitant NSSI group tended to occur in early adolescence. In contrast, the age of onset in the nonconcomitant NSSI group tended to occur in late adolescence. The concomitant NSSI group had significantly higher CTQ‐SF, HAMD, HAMA, and TAS scores than the nonconcomitant NSSI group (all *p* < .01).

**TABLE 1:** Comparison of demographic characteristics and CTQ‐SF, HAMD, HAMA, and TAS‐20 scores of the concomitant NSSI and nonconcomitant NSSI groups

	NSSI	Non‐NSSI		
Variables	(*N* = 112)	(*N* = 72)	*χ* ^2^/*t*	*p*
Sex, *N* (%)			3.093	.079
Male	34 (30.36)	31 (43.06)		
Female	78 (69.64)	41 (56.94)		
Only child, *N* (%)			0.617	.432
Yes	29 (25.89)	15 (20.83)		
No	83 (74.11)	57 (79.17)		
Residence, *N* (%)			0.669	.413
Rural	31 (27.68)	24 (33.33)		
Urban	81 (72.32)	48 (66.67)		
Age of onset, *N* (%)			20.516	<.01
12–13	34 (30.36)	4 (5.56)		
14–15	39 (34.82)	23 (31.94)		
16–18	39 (34.82)	45 (62.50)		
CTQ‐SF (*M* ± SD)	56.54 ± 13.8	45.92 ± 11.72	5.4	<.01
HAMD (*M* ± SD)	36.83 ± 8.31	30.07 ± 9.13	5.181	<.01
HAMA (*M* ± SD)	26.68 ± 8.57	19.9 ± 7.18	5.567	<.01
TAS‐20 (*M* ± SD)	69.04 ± 7.64	63.96 ± 8.94	4.123	<.01

CTQ‐SF, Childhood Trauma Questionnaire‑Short Form; HAMD, Hamilton Depression Scale; HAMA, Hamilton Anxiety Scale; TAS‐20, Toronto Alexithymia Scale.

### 3.2 Correlation analysis of the ANSAQ, CTQ‐SF, HAMD, HAMA, and TAS‐20 scores in the concomitant NSSI group

Correlation analysis showed in Table 2 that childhood trauma was significantly and positively correlated with self‐injury, depression, and anxiety for all subscales (all *p* < .05), except sexual abuse. Difficulty with emotion recognition and self‐injury were significantly and positively correlated (*p* < .01), Alexithymia was significantly and positively correlated with depression and anxiety for all subscales (all *p* < .01).

**TABLE 2:** Correlation analysis of the ANSAQ, CTQ‐SF, HAMD, HAMA, and TAS‐20 scores in the concomitant NSSI group

	1	2	3	4	5	6	7	8	9	10	11
ANSAQ	1										
EA	.439**	1									
PA	.331**	.471**	1								
SA	.074	.241*	.143	1							
EN	.358**	.505**	.277**	.060	1						
PN	.270**	.480**	.345**	.177	.476**	1					
DDF	.045	.090	.098	.049	.016	.044	1				
DIF	.271**	.307**	.127	.117	.035	.102	.579**	1			
EOT	.087	.060	.164	.020	.162	.305**	.036	.012	1		
HAMD	.452**	.523**	.286**	.125	.363**	.323**	.244**	.503**	.304**	1	
HAMA	.482**	.433**	.243**	.037	.215*	.285**	.295**	.609**	.311**	.753**	1

ANSAQ, Adolescent Nonsuicidal Self‐Injury Assessment Questionnaire; EA, emotional abuse; PA, physical abuse; SA, sexual abuse; EN, emotional neglect; PN, physical neglect; DDF, difficulty describing feelings; DIF, difficulty identifying feelings; EOT, externally oriented thinking; HAMD, Hamilton Depression Scale; HAMA, Hamilton Anxiety Scale.

* *p* < .05.

** *p* < .01.

### 3.3 Risk factor analysis for NSSI

Logistic regression analysis with NSSI and non‐NSSI as dependent variables and sex, age of onset, childhood trauma, alexithymia, depression, and anxiety as independent variables. The model is significant (*χ* ^2^ = 62.11, *p* < .01) and account for 28.7% (Cox and Snell R^2^) to 38.8% (Nagelkerke *R* ^2^) of the variance. The results in Table 3 show that emotional abuse (*p* = .028, OR = 1.14, 95% CI = 1.01–1.28) and age of onset (*p* = .009) were risk factors for NSSI. Adolescents with onset age 12–13 years (OR = 6.30, 95% CI = 1.72–23.10) and 14–15 years (OR = 2.24, 95% CI = 1.04–4.84) had a higher risk of self‐injury relative to adolescents aged 16–18 years.

**TABLE 3:** Risk factor analysis for NSSI

Variables	*B*	SE	Wald	*p*	OR (95% CI)
EA	0.13	0.06	4.84	.028	1.14 (1.01–1.28)
PA	–0.01	0.08	0.02	.902	0.99 (0.84–1.16)
SA	0.21	0.17	1.40	.237	1.23 (0.87–1.72)
EN	–0.02	0.05	0.15	.695	0.98 (0.89–1.08)
PN	0.01	0.07	0.01	.914	1.01 (0.88–1.15)
DDF	–0.04	0.08	0.30	.584	0.96 (0.82–1.12)
DIF	0.06	0.07	0.87	.351	1.07 (0.93–1.22)
EOT	0.05	0.05	1.04	.308	1.05 (0.95–1.17)
HAMD	0.01	0.03	0.14	.712	1.01 (0.95–1.08)
HAMA	0.04	0.04	1.14	.285	1.04 (0.97–1.11)
Sex	0.24	0.38	0.38	.535	1.27 (0.60–2.70)
Age of onset^a^			9.50	.009	
12–13	1.84	0.66	7.70	.006	6.30 (1.72–23.10)
14–15	0.81	0.39	4.25	.039	2.24 (1.04–4.84)
Constant	–6.13	1.83	11.22	.001	0.00

^a^ 16–18 years old as the reference group.

EA, emotional abuse; PA, physical abuse; SA, sexual abuse; EN, emotional neglect; PN, physical neglect; DDF, difficulty describing feelings; DIF, difficulty identifying feelings; EOT, externally oriented thinking; HAMD, Hamilton Depression Scale; HAMA, Hamilton Anxiety Scale.

## 4 DISCUSSION

The results showed that the age of onset in the NSSI group occurred more often in early adolescence compared with the non‐NSSI group. On the one hand, this finding may be related to the imperfect development of the prefrontal cortex in early adolescence, which is prone to NSSI (Moran et al., 2012). On the other hand, it may be related to the formation of identity. Adolescence is in the stage of identity formation, and identity synthesis is the process of constructing ideals, values, and goals of a person's identity by adjusting childhood identity. Identity confusion can occur if the goals and commitments of adolescent self‐identity are not formed. Identity synthesis may promote the formation of a positive self and interpersonal relationships, whereas identity confusion may increase mood swings (Schwartz et al., 2009). Early adolescence is prone to NSSI, and it may be that identity confusion is most severe during this period. One study has reported that NSSI was negatively correlated with identity synthesis and positively correlated with identity confusion, and that NSSI may be a means used by adolescents to cope with identity confusion (Claes et al., 2014). The results showed that the NSSI group had significantly higher scores in childhood trauma, alexithymia, depression, and anxiety than the non‐NSSI group. Several studies have also found a strong association between NSSI and childhood trauma, alexithymia, depression, and anxiety (Groschwitz et al., 2015; Hu et al., 2021; Norman & Borrill, 2015; Thomassin et al., 2016).

The correlation analysis results were contrary to previous findings, such as those of Thomassin et al. (2016) who reported a correlation between sexual abuse and NSSI in a sample of adolescent hospitalizations. This difference may be due to cultural causes. In traditional Chinese culture, people have conservative attitudes toward sex and are usually reluctant to discuss it openly. In this population, sex is often combined with shame and disgrace and is stigmatized. As a result, some people may choose to hide their experiences of sexual abuse to avoid negative comments and pressure from others (Liu et al., 2021). This study supports the idea that childhood abuse is positively associated with self‐injurious behavior, including emotional abuse, physical abuse, and neglect (Paul & Ortin, 2019). The development of emotion regulation skills is hindered by ineffective family environment during childhood, which may include parental abuse and neglect, parental denial of the legitimacy of emotional expression, and lack of support for emotional expression by the child. In particular, emotional abuse may disrupt an individual's ability to regulate negative emotions. In the absence of positive resilience to negative emotions, individuals tend to employ dysfunctional coping mechanisms, such as the use of NSSI, to alleviate these distressing emotions (Kooiman et al., 2004; Wang et al., 2022).

The present study reflects the view of Norman and Borrill (2015); that is, the difficulty in identifying feelings (DIF) is significantly and positively associated with self‐injury. However, previous studies have shown contrary findings regarding the nature of the relationship between alexithymia and self‐harm over time. Anderson and Crowther (2012) found that undergraduate students who had recent or previously engaged in self‐harm had significantly higher scores of DIF than those who had not self‐harmed and that the DIF scores between the two groups had no significant difference. However, Moseley et al. (2019) found that in alexithymia, participants who had not self‐injured were difficult to distinguish from those who had a history of self‐injury. This finding may be related to the less stable nature of alexithymia that change over time (Iskric et al., 2020). Central to alexithymia is the difficulty in identifying and describing emotions. From the perspective of multiple coding theory, emotional information processing is the interconnection of nonverbal subsymbolic content (i.e., sensory, visceral, kinesthetic, and motor activity in states of emotional arousal), nonverbal symbolic content (images), and verbal symbolic content (words) through a referential process. Alexithymia is considered to be a disruption of this referential process, allowing the separation of verbal and nonverbal systems (Bucci, 2007). Due to limited tolerance and emotional perception in patients with alexithymia, inhibitory regulation strategies, such as NSSI, are often used to reduce emotional arousal when confronted with strong negative emotions.

Regression analysis shows that emotional abuse and age of onset are associated with risk of NSSI, and this result was consistent with previous studies (Thomassin et al., 2016). According to the World Health Organization, emotional abuse (36.3%) is more common than physical abuse (22%), neglect (16.3%), and sexual abuse (18% of girls and 8% of boys) globally (World Health Organization, & International Society for Prevention of Child Abuse & Neglect, 2006). Emotional abuse is mainly reflected in negative interactions between parent–child relationships, including any words or actions that criticize or humiliate the child. Due to a prolonged childhood environment of emotional failure, emotional needs are not effectively responded to. Such phenomenon often alters the individual's perception of self and external reality, develops a self‐critical cognitive style, and generates feelings of self‐blame and helplessness. When faced with unpleasant emotional experiences, individuals may use NSSI as a means of self‐punishment and try to replace feelings of despair with illusions of self‐control to avoid emotional pain and obtain temporary relief and relaxation (Tatnell et al., 2017).

The results of this study show a trend of decreasing NSSI from early to late adolescence, which has also been found in previous studies (Wilkinson et al., 2022). The increase in NSSI in early adolescence may be associated with impulsivity and strong emotional responses during this period, hormonal influences, and possible exposure to infectious NSSI behaviors among adolescent peers (Jarvi et al., 2013). In contrast, in late adolescence, probably because of the prefrontal cortex development, emotions are controlled, and NSSI behaviors are reduced. The choice of emotion regulation strategy may be influenced by age, and adolescents engage in emotion regulation through the NSSI. Studies have shown that the neural circuitry between emotional processing and pain processing is closely linked, that the offset of physical pain leads to improved mood, and that the anticipation of improved mood causes alterations in μ‐opioid receptor activity in brain regions. Thus, people who experience self‐injury associate pain offset with mood improvement, and then increased anticipation of reward after NSSI further reinforces NSSI behavior (Navratilova & Porreca, 2014; van der Venne et al., 2021).

In summary, this study explored the relationship between childhood trauma, alexithymia, and NSSI in adolescents with BD II depression. The results showed that NSSI was associated with early childhood trauma, particularly emotional abuse. Childhood trauma and narrative disorders, as risk factors affecting the development of physical and mental health in children and adolescents, have important implications in the assessment and intervention of NSSI and BD II depression. This study have some limitations that need to be addressed: (1) the authenticity of retrospective reports of childhood trauma was questionable due to emotional and recall biases; (2) the use of self‐reports may hinder the effective assessment of the emotional deficits that patients with alexithymia are not aware of themselves due to their lack of emotional introspection (Luca et al., 2013); (3) this study was a cross‐sectional survey, and no inference of causal conclusions could be made; (4) the sample size of this study was not large enough, and the representativeness of the results may be limited; (5) the timing and frequency of NSSI occurrence were imprecise. Future research on the causal mechanisms of childhood trauma and alexithymia on the occurrence of NSSI in patients with BP II requires the use of longitudinal studies to clarify this issue.

## 5 CONCLUSION

Childhood trauma and alexithymia were significant factors of NSSI in patients with BD II depression. Emotional abuse and age of onset as risk factors for NSSI, and difficulties in emotion recognition were positively associated with the patients’ NSSI. This study identifies emotional abuse and emotional recognition difficulties as key to preventing and intervening in adolescents' NSSI, providing a safe and stable home atmosphere for children, improving adolescents' emotional regulation skills such as mindfulness, and helping them become aware of their emotions so that they can adopt appropriate coping strategies to reduce NSSI behaviors.

## CONFLICT OF INTEREST

No potential conflict of interest was reported by the authors.

### PEER REVIEW

The peer review history for this article is available at: https://publons.com/publon/10.1002/brb3.2771.

## Data Availability

The data that support the findings of this study are available from the corresponding author upon reasonable request.

## References

[brb32771-bib-0001] Aas, M. , Henry, C. , Andreassen, O. A. , Bellivier, F. , Melle, I. , & Etain, B. (2016). The role of childhood trauma in bipolar disorders. International Journal of Bipolar Disorders, 4(1), 1–10. 26763504 10.1186/s40345-015-0042-0 PMC4712184

[brb32771-bib-0002] Anderson, N. L. , & Crowther, J. H. (2012). Using the experiential avoidance model of non–suicidal self‐injury: Understanding who stops and who continues. Archives of Suicide Research, 16(2), 124–134. 22551043 10.1080/13811118.2012.667329

[brb32771-bib-0003] Bucci, W. (2007). Dissociation from the perspective of multiple code theory, part I: Psychological roots and implications for psychoanalytic treatment. Contemporary Psychoanalysis, 43(2), 165–184.

[brb32771-bib-0004] Chen, J. J. , Xie, J. , Zeng, L. , Zhou, C. J. , Zheng, P. , & Xie, P. (2019). Urinary metabolite signature in bipolar disorder patients during depressive episode. Aging (Albany NY), 11(3), 1008. 30721880 10.18632/aging.101805 PMC6382435

[brb32771-bib-0005] Claes, L. , Luyckx, K. , & Bijttebier, P. (2014). Non‐suicidal self‐injury in adolescents: Prevalence and associations with identity formation above and beyond depression. Personality and Individual Differences, 61, 101–104.

[brb32771-bib-0006] Diagnostic and Statistical Manual of Mental Disorders (2013). 5th edn. (DSM‐5). Arlington VA, American Psychiatric Association.

[brb32771-bib-0007] Groschwitz, R. C. , Plener, P. L. , Kaess, M. , Schumacher, T. , Stoehr, R. , & Boege, I. (2015). The situation of former adolescent self‐injurers as young adults: A follow‐up study. Bmc Psychiatry [Electronic Resource], 15(1), 1–9. 10.1186/s12888-015-0555-1 PMC4506399 26187150

[brb32771-bib-0008] Hu, Z. , Yu, H. , Zou, J. , Zhang, Y. , Lu, Z. , & Hu, M. (2021). Relationship among self‐injury, experiential avoidance, cognitive fusion, anxiety, and depression in Chinese adolescent patients with non‐suicidal self‐injury. Brain and Behavior, 11(12), e2419. 34816613 10.1002/brb3.2419 PMC8671785

[brb32771-bib-0009] International Society for the Study of Self‐injury . (2018). What is self‐injury? https://itriples.org/about‐self‐injury/what‐is‐self‐injury/ (accessed Oct 1, 2019)

[brb32771-bib-0010] Iskric, A. , Ceniti, A. K. , Bergmans, Y. , McInerney, S. , & Rizvi, S. J. (2020). Alexithymia and self‐harm: A review of non‐suicidal self‐injury, suicidal ideation, and suicide attempts. Psychiatry Research, 288, 112920. 32279008 10.1016/j.psychres.2020.112920

[brb32771-bib-0011] Jarvi, S. , Jackson, B. , Swenson, L. , & Crawford, H. (2013). The impact of social contagion on non‐suicidal self‐injury: A review of the literature. Archives of Suicide Research, 17, 1–19. 23387399 10.1080/13811118.2013.748404

[brb32771-bib-0012] Kang, N. , Jiang, Y. , Ren, Y. , Gong, T. , Liu, X. , Leung, F. , & You, J. (2018). Distress intolerance mediates the relationship between child maltreatment and non‐suicidal self‐injury among Chinese adolescents: A three‐wave longitudinal study. Journal of Youth and Adolescence, 47(10), 2220–2230. 29942987 10.1007/s10964-018-0877-7

[brb32771-bib-0013] Kerner, B. (2014). Genetics of bipolar disorder. The Application of Clinical Genetics, 7, 33. 24683306 10.2147/TACG.S39297 PMC3966627

[brb32771-bib-0014] Kooiman, C. G. , van Rees Vellinga, S. , Spinhoven, P. , Draijer, N. , Trijsburg, R. W. , & Rooijmans, H. G. (2004). Childhood adversities as risk factors for alexithymia and other aspects of affect dysregulation in adulthood. Psychotherapy and Psychosomatics, 73(2), 107–116. 14767153 10.1159/000075542

[brb32771-bib-0015] Lim, K. S. , Wong, C. H. , McIntyre, R. S. , Wang, J. , Zhang, Z. , Tran, B. X. , Tan, W. , Ho, C. S. , & Ho, R. C. (2019). Global lifetime and 12‐month prevalence of suicidal behavior, deliberate self‐harm and non‐suicidal self‐injury in children and adolescents between 1989 and 2018: A meta‐analysis. International Journal of Environmental Research and Public Health, 16(22), 4581. 10.3390/ijerph16224581 PMC6888476 31752375

[brb32771-bib-0016] Lindquist, K. A. , Satpute, A. B. , Wager, T. D. , Weber, J. , & Barrett, L. F. (2016). The brain basis of positive and negative affect: Evidence from a meta‐analysis of the human neuroimaging literature. Cerebral Cortex, 26(5), 1910–1922. 25631056 10.1093/cercor/bhv001 PMC4830281

[brb32771-bib-0017] Liu, H. , Wang, W. , Yang, J. , Guo, F. , & Yin, Z. (2021). The effects of alexithymia, experiential avoidance, and childhood sexual abuse on non‐suicidal self‐injury and suicidal ideation among Chinese college students with a history of childhood sexual abuse. Journal of Affective Disorders, 282, 272–279. 33418378 10.1016/j.jad.2020.12.181

[brb32771-bib-0018] Luca, M. , Luca, A. , & Calandra, C. (2013). Psychomotor retardation and externally oriented thinking in major depression. Neuropsychiatric Disease and Treatment, 9, 759. 23745046 10.2147/NDT.S44650 PMC3671799

[brb32771-bib-0019] Miller, S. , Dell'Osso, B. , & Ketter, T. A. (2014). The prevalence and burden of bipolar depression. Journal of Affective Disorders, 169, S3–S11. 25533912 10.1016/S0165-0327(14)70003-5

[brb32771-bib-0020] Moran, P. , Coffey, C. , Romaniuk, H. , Olsson, C. , Borschmann, R. , Carlin, J. B. , & Patton, G. C. (2012). The natural history of self‐harm from adolescence to young adulthood: A population‐based cohort study. The Lancet, 379(9812), 236–243. 10.1016/S0140-6736(11)61141-0 22100201

[brb32771-bib-0021] Moseley, R. L. , Gregory, N. J. , Smith, P. , Allison, C. , & Baron‐Cohen, S. J. M. A. (2019). A ‘choice’, an ‘addiction’, a way ‘out of the lost’: Exploring self‐injury in autistic people without intellectual disability. Molecular Autism, 10(1), 1–23. 31007885 10.1186/s13229-019-0267-3 PMC6458651

[brb32771-bib-0022] Navratilova, E. , & Porreca, F. (2014). Reward and motivation in pain and pain relief. Nature Neuroscience, 17(10), 1304–1312. 25254980 10.1038/nn.3811 PMC4301417

[brb32771-bib-0023] Norman, H. , & Borrill, J. (2015). The relationship between self‐harm and alexithymia. Scandinavian Journal of Psychology, 56(4), 405–419. 26011069 10.1111/sjop.12217

[brb32771-bib-0024] Paquola, C. , Bennett, M. R. , & Lagopoulos, J. (2016). Understanding heterogeneity in grey matter research of adults with childhood maltreatment—A meta‐analysis and review. Neuroscience & Biobehavioral Reviews, 69, 299–312. 27531235 10.1016/j.neubiorev.2016.08.011

[brb32771-bib-0025] Paul, E. , & Ortin, A. (2019). Psychopathological mechanisms of early neglect and abuse on suicidal ideation and self‐harm in middle childhood. European Child & Adolescent Psychiatry, 28(10), 1311–1319. 30783774 10.1007/s00787-019-01287-8

[brb32771-bib-0026] Ribeiro, J. D. , Franklin, J. C. , Fox, K. R. , Bentley, K. H. , Kleiman, E. M. , Chang, B. P. , & Nock, M. K. (2016). Self‐injurious thoughts and behaviors as risk factors for future suicide ideation, attempts, and death: A meta‐analysis of longitudinal studies. Psychological Medicine, 46(2), 225–236. 26370729 10.1017/S0033291715001804 PMC4774896

[brb32771-bib-0027] Rybakowski, J. K. (2017). Recent advances in the understanding and management of bipolar disorder in adults. F1000Research, 6, . 10.12688/f1000research.12329.1 PMC5698915 29188027

[brb32771-bib-0028] Schwartz, S. J. , Zamboanga, B. L. , Wang, W. , & Olthuis, J. V. (2009). Measuring identity from an Eriksonian perspective: Two sides of the same coin. Journal of Personality Assessment, 91(2), 143–154. 19205935 10.1080/00223890802634266

[brb32771-bib-0029] Serafini, G. , Pompili, M. , Borgwardt, S. , Houenou, J. , Geoffroy, P. A. , Jardri, R. , Girardi, P. , & Amore, M. (2014). Brain changes in early‐onset bipolar and unipolar depressive disorders: A systematic review in children and adolescents. European Child & Adolescent Psychiatry, 23(11), 1023–1041. 10.1007/s00787-014-0614-z. Epub 2014 Sep 12. PMID: 25212880. 25212880

[brb32771-bib-0030] Solano, P. , Ustulin, M. , Pizzorno, E. , Vichi, M. , Pompili, M. , Serafini, G. , & Amore, M. (2016). A Google‐based approach for monitoring suicide risk. Psychiatry Research, 246, 581–586. 10.1016/j.psychres.2016.10.030. Epub 2016 Oct 20. PMID: 27837725. 27837725

[brb32771-bib-0031] Sun, D. , Zhang, R. , Ma, X. , Sultana, M. S. , Jiao, L. , Li, M. , Liu, Q. , & Li, Z. (2022). The association between childhood trauma and the age of onset in drug‐free bipolar depression. Psychiatry Research, 310, 114469. 35231875 10.1016/j.psychres.2022.114469

[brb32771-bib-0032] Tatnell, R. , Hasking, P. , Newman, L. , Taffe, J. , & Martin, G. (2017). Attachment, emotion regulation, childhood abuse and assault: Examining predictors of NSSI among adolescents. Archives of Suicide Research, 21(4), 610–620. 27726519 10.1080/13811118.2016.1246267

[brb32771-bib-0033] Thomassin, K. , Shaffer, A. , Madden, A. , & Londino, D. L. (2016). Specificity of childhood maltreatment and emotion deficit in non‐suicidal self‐injury in an inpatient sample of youth. Psychiatry Research, 244, 103–108. 27479099 10.1016/j.psychres.2016.07.050

[brb32771-bib-0034] Van der Venne, P. , Balint, A. , Drews, E. , Parzer, P. , Resch, F. , Koenig, J. , & Kaess, M. (2021). Pain sensitivity and plasma beta‐endorphin in adolescent non‐suicidal self‐injury. Journal of Affective Disorders, 278, 199–208. 32961416 10.1016/j.jad.2020.09.036

[brb32771-bib-0035] Wan, Y. H. , Liu, W. , Hao, J. H. , & Tao, F. B. (2018). Development and evaluation on reliability and validity of adolescent non‐suicidal self‐injury assessment questionnaire. Chinese Journal of School Health, 39(2), 170–173.

[brb32771-bib-0036] Wang, Y. J. , Li, X. , Ng, C. H. , Xu, D. W. , Hu, S. , & Yuan, T. F. (2022). Risk factors for non‐suicidal self‐injury (NSSI) in adolescents: A meta‐analysis. EClinicalMedicine, 46, 101350. 35330803 10.1016/j.eclinm.2022.101350 PMC8938878

[brb32771-bib-0037] Wang, Y. L. , Qin, Y. L. , Xiao, C. , & Lin, X. Y. (2016). The relationship between interparental conflict and adolescents’ self‐injury: A moderate mediation model. Psychological Development and Education, 32(3), 377–384.

[brb32771-bib-0038] Weintraub, M. J. , Van de Loo, M. M. , Gitlin, M. J. , & Miklowitz, D. J. (2017). Self‐harm, affective traits, and psychosocial functioning in adults with depressive and bipolar disorders. The Journal of Nervous and Mental Disease, 205(11), 896. 29077652 10.1097/NMD.0000000000000744 PMC5679240

[brb32771-bib-0039] Wilkinson, P. O. , Qiu, T. , Jesmont, C. , Neufeld, S. A. , Kaur, S. P. , Jones, P. B. , & Goodyer, I. M. (2022). Age and sex effects on non‐suicidal self‐injury, and their interplay with psychological distress. Journal of Affective Disorders, 306, 240–245. 35304237 10.1016/j.jad.2022.03.021

[brb32771-bib-0040] World Health Organisation (2014). Global status report on violence prevention 2014. Geneva: Retrieved from http://www.who.int/violence_injury_prevention/violence/statusreport/2014/en/

[brb32771-bib-0041] World Health Organization, & International Society for Prevention of Child Abuse and Neglect (2006). Preventing Child Maltreatment: A guide to taking action and generating evidence. Geneva.

[brb32771-bib-0042] Yi, J. Y. , Yao, S. Q. , & Zhu, X. Z. (2003). Reliability and validity analysis of the Chinese version of the TAS‐20. Chinese Journal of Mental Health, 17(11), 763–767.

[brb32771-bib-0043] Zetterqvist, M. , Lundh, L. G. , Dahlström, Ö. , & Svedin, C. G. (2013). Prevalence and function of non‐suicidal self‐injury (NSSI) in a community sample of adolescents, using suggested DSM‐5 criteria for a potential NSSI disorder. Journal of Abnormal Child Psychology, 41(5), 759–773. 23344701 10.1007/s10802-013-9712-5

[brb32771-bib-0044] Zhao, X. , Zhang, Y. , Li, Y. , Zhou, Y. , Li, H. , & Yang, S. (2005). Reliability and validity of Chinese version of childhood trauma questionnaire. Chinese Journal of Clinical Rehabilitation, 9(20), 105–107.

